# Effect of a functional brace in combination with physical therapy for early correction of cubitus varus in young children

**DOI:** 10.1186/s12887-022-03578-7

**Published:** 2022-09-03

**Authors:** Qiang Shi, Hua Yan, Shu Chen, Qian Cao, Yuxia Xu

**Affiliations:** 1 Department of Spine Surgery, The Affiliated Changsha Central Hospital, Hengyang Medical School, University of South China, Changsha, 410018 People’s Republic of China; 2 Department of Pediatric Orthopedics, Shantou University Guangzhou Huaxin Orthopedic Hospital, Guangzhou, 510507 People’s Republic of China; 3 Department of Orthopedics, Xiangtan Chinese Medicine Hospital, Xiangtan, 411100 People’s Republic of China

**Keywords:** Cubitus varus, Functional brace, Physical therapy, Paediatric

## Abstract

**Background:**

This study aimed to assess the clinical and radiologic outcomes of a functional brace in combination with physical therapy (FBPT) for early correction of cubitus varus in young children.

**Methods:**

Eighteen consecutive patients with cubitus varus secondary to supracondylar fractures were enrolled between July 2017 and March 2019. We used the FBPT technique to correct varus and sagittal plane deformity for early cubitus varus in young children. The clinical evaluation included measurement of varus angulation, sagittal plane, and range of motion at three, six, and twelve months post-intervention. The clinical and radiographic results were assessed according to the Bellemore criteria.

**Results:**

Pre-treatment humerus-elbow-wrist (HEW) angle measured on the affected side (varus deformity) ranged between -38° and -12° (average, -23.2°) while the post-treatment HEW angle ranged between -10° and + 15° (average, 8.8°). Compared with the unaffected side, no statistically significant difference was found in the affected side post-intervention (*P* > 0.05). According to the Bellemore criteria, we got excellent results in fourteen patients (77.8%), good results in three patients (16.7%), and poor result in one patient (5.5%). All patients and their parents (except one patient with residual varus deformities) were satisfied with the functional and cosmetic outcomes.

**Conclusions:**

The FBPT is effective for the treatment of cubitus varus in children, especially for young children within 6 months of the injury.

## Introduction

Cubitus varus is the most common delayed complication of Gartland type III supracondylar humeral fracture, which belongs to complex 3-dimensional (3D) deformity including varus, internal rotation, and hyperextension deformities of the elbow joint [1–3]. Owing to lack of the growth potential around the elbow, spontaneous correction of the cubitus varus is highly unlikely in children [4]. Until now, various surgical techniques for cubitus varus correction have been recommended, such as distal humeral osteotomy with cross-pin fixation, plating, as well as external fixation [5–7]. However, no gold standard technique can achieve patient satisfaction for ideal cosmetic appearance while minimizing complications [8].

The ultimate goal for cubitus varus correction is to gain good functional and cosmetic outcomes. Nevertheless, over 20% complication rates for corrective osteotomies of the distal humerus have been reported in the past decades [9–12]. Oppenheim et al. reported various post-operative complications after performing 45 corrective supracondylar osteotomies, including sepsis, neurapraxia, and unacceptable scarring [10]. Ippolito et al. performed supracondylar osteotomies for cubitus varus correction with 24% immediate complication rates, such as hematoma, ulnar-nerve palsy, or circulatory disturbance [12]. To date, rapid development in surgical techniques and implants has resulted in a remarkable increase in the surgical treatment of cubitus varus deformity, however, no conservative treatment of cubitus varus deformity has been reported.

In this study, we report the radiological and clinical outcome of a new functional brace in combination with physical therapy (FBPT) for early correction of cubitus varus in young children (ages 2–8 years old). First, the novel technique is simple and achieves good functional and cosmetic outcomes with minimal complications which might also help mitigate the cost and risk of surgery. Second, iatrogenic ulnar nerve injury or elbow stiffness occurs during the procedure of osteotomy with cross pinning sometimes while this nonoperative method avoids lateral prominences or skin scars usually present following closing wedge osteotomy, allowing for better cosmesis.

## Materials and methods

Eighteen consecutive patients with post-traumatic cubitus varus deformity were enrolled in our hospital during the time between July 2017 and March 2019. This monocentric retrospective study evaluated the clinical and radiologic outcomes of FBPT for early correction of cubitus varus in young children. Clinically cubitus varus is assessed by measuring the carrying angle of the arm. This is the angle created by the medial border of the fully supinated forearm and medial border of the humerus, with the elbow extended (5–10 degrees) [13]. Individuals with elbow contractures, previous neurovascular injuries, and burn scars over the elbow were excluded in the present study. Informed consent was acquired from all parents and the institutional ethical committee of Changsha Central Hospital, University of South China approved this study. Analyses and procedures performed in the study were conducted in accordance with the Declaration of Helsinki [14].

The inclusion criteria: 1- 15 degrees < Cubitus varus deformity < 40 degrees, 2- Ages 2–8 years old, 3- Malunion of supracondylar humerus fracture, 4- Follow up more than 24 months post-operative. 5- Indicated for surgery due to functional limitation or secondary to poor cosmesis.

All the patients sustained supracondylar humeral fracture at the time of injury. Six patients received closed reduction and K-wire fixation while twelve received previous conservative treatment. Patient’s pre- and post-operative parameters are listed in Table 1. Images was performed after informed consent was obtained from the parents.

**Table 1:** Patient’s pre- and post-treatment parameters

Case	Age (year)	Sex	Side	Time since injury (month)	Follow up (month)	HEW angle (°)			Flexion/extension (maximum)		Bellemore criteria	
						Pre-treatment	Post-treatment	Normal	Pre-treatment	Post-treatment	Pre-treatment	Post-treatment
1	5	M	R	5	25	-12	8	6	143/0	143/0	poor	excellent
2	4	F	L	3	26	-22	9	8	135/0	138/0	poor	excellent
3	6	M	L	4	34	-23	15	7	134/6	135/4	poor	excellent
4	7	M	R	9	30	-25	8	9	128/0	134/0	poor	good
5	8	F	L	11	28	-28	-10	8	121/10	127/8	poor	poor
6	2	M	R	3	40	-19	9	7	139/0	139/0	poor	excellent
7	3	M	R	4	42	-22	7	9	134/8	136/6	poor	excellent
8	6	F	L	8	39	-25	15	8	140/0	141/0	poor	excellent
9	8	F	R	6	31	-26	3	9	130/3	133/0	poor	good
10	3	M	L	2	25	-21	11	10	137/0	139/0	poor	excellent
11	7	F	R	10	29	-23	6	11	133/0	138/0	poor	good
12	5	M	R	4	52	-18	11	9	142/8	143/4	poor	excellent
13	4	M	L	3	38	-18	12	7	134/0	134/0	poor	excellent
14	3	M	L	3	27	-26	13	8	138/0	138/0	poor	excellent
15	5	F	L	2	30	-21	15	7	135/0	135/0	poor	excellent
16	3	M	R	4	34	-26	8	8	119/0	132/0	poor	excellent
17	3	F	L	2	37	-38	9	10	130/11	140/6	poor	excell
18	5	F	R	3	34	-25	10	8	137/5	139/2	poor	excellent
Variable	4 ± 2			5 ± 3	33 ± 7	-23 ± 5	9 ± 6	8 ± 1	137 ± 3/2 ± 4	138 ± 3/2 ± 0		

The clinical and radiographic outcomes were assessed according to the Bellemore criteria [15] at the last follow-up. The blinded assessor, who was not involved in the intervention, then conducted the pre- and post-treatment assessments.

### Conservative treatment protocol

**Fig. 1:**
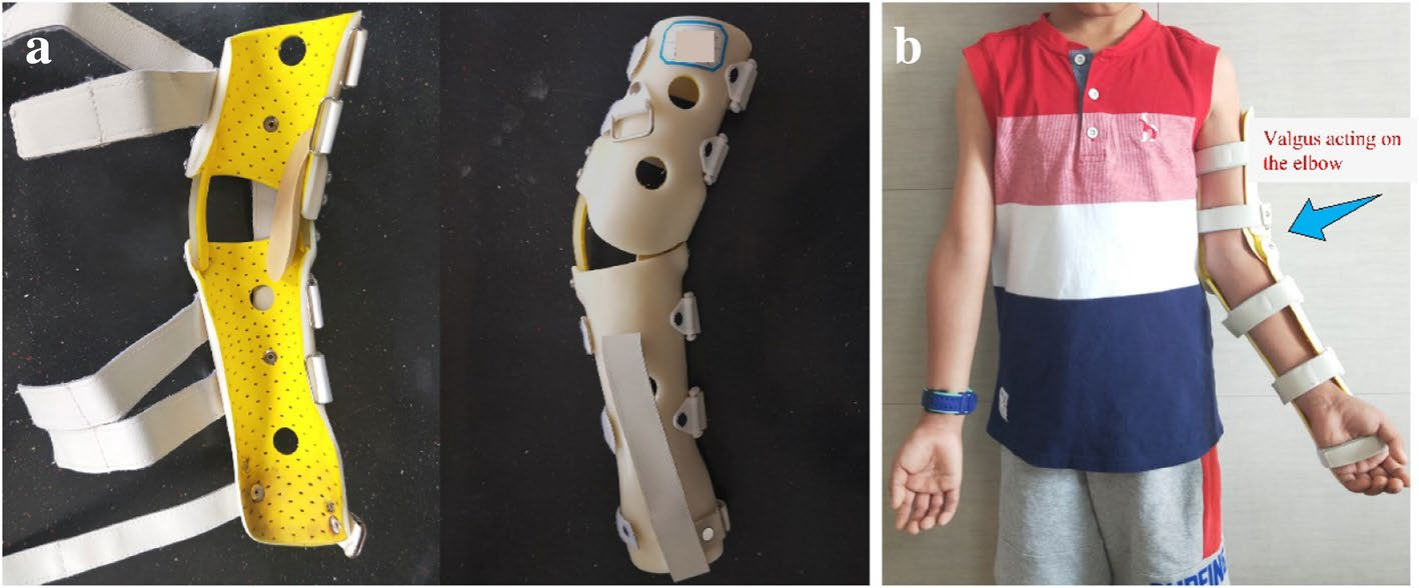
**a** The photographs of a novel functional brace. **b** Mechanisms and effects of the functional brace for early correction of cubitus varus in young children

**Fig. 2:**
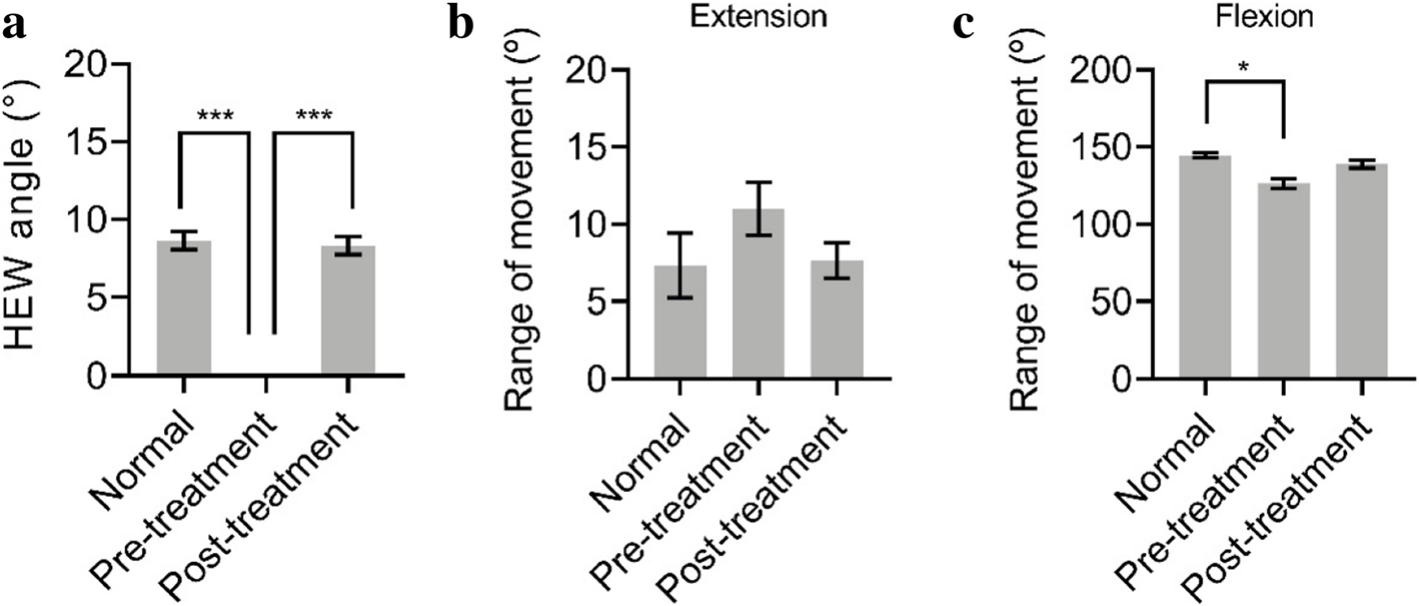
The clinical and radiographic results were assessed according to the Bellemore criteria. **a** Changes in the HEW angles after FBPT compared with the unaffected arm and the normal side. **b** The range of movement of the elbow joint is almost the same as for the normal arm in extension. **c** Compared with the normal side, statistically significant difference was found for the range of movement of the elbow joint in pre-treatment flexion. * *P* < 0.05, *** *P* < 0.001

The conservative treatment with FBPT protocol is based on the following guidelines: (1) all patients in this study are asked to wear a functional brace (valgus > 30 degrees) with the elbow at 90° of flexion from the diagnosis day, then tighten the commercial sling in the extension and supination position for 3 months (Fig. 1); (2) the sling was removed one hour every day for range-of-motion elbow exercises with assistance from their physiotherapist to increase valgus load on the elbow joint. These exercises consisted of elbow extension with the forearm in full supination, as well as valgus exercises at maximum extent, as tolerated by the patient. Each exercise therapy was applied for 30 s in the extension and supination position followed by 20 s of relaxation. Each set of physical therapy was repeated 20 repetitions 3 times a day; (3) from 3 to 12 months, the patients were required to wear the functional brace 10–12 h during the night time. A physical therapy program was also implemented at this stage both in extension and valgus position, in sets of 50 repetitions 10 times a day. (4) after 12 months, if the deformity has achieved complete correction, patients wear the functional brace for another 3 months 8–10 h during the night time; if not, we will continue the FBPT until complete correction of cubitus varus.

### Evaluation of post-treatment

Radiographic evaluation was performed at month 3 to 6 during post initial surgery to observe the changes of cubitus varus. Radiographic evaluation included the humerus-elbow-wrist (HEW) angle on the anteroposterior radiograph of the elbow while clinical evaluations included active and passive range of elbow joint in the outpatient clinic. After the conservative treatment with FBPT, the HEW angle, active and passive range of elbow joint were reviewed at 1, 3, 6, and 12 months and then every year until skeletal maturity. The results were assessed according to the Bellemore criteria at the last follow-up.

### Statistical analysis

Data analysis of our samples (pre-treatment and post-treatment at last follow-up) were compared using the Student t-test. The continuous variables were expressed as mean ± standard deviation. For statistical analysis, SPSS software (version 22.0; IBM, Armonk, NY, USA) was performed. The level of significance was set at *p* < 0.05. Power calculation was used in all cases.

## Results

A total of 10 male (55%) and 8 female (45%) patients were treated conservatively with FBPT and assessed after a mean follow-up period of 33 ± 8 months (range, 25–42 months). The mean period between initial injury and FBPT was 5 ± 3 months (range, 2–12 months) in this study.

### Radiographic outcome

Pre-treatment HEW angle measured on the affected side (varus deformity) ranged between -38° and -12° (average, -23°) while the post-treatment HEW angle ranged between -10° and + 15° (average, 9°) (Table 1). There was no significantly difference at post-treatment between the affected side and the normal side (*p* = 0.6896). However, compared with the pre-treatment HEW angle, statistically significant difference was found in the post-treatment HEW angle on the affected side (*p* < 0.001) (Fig. 2).

### Clinical outcome

**Fig. 3:**
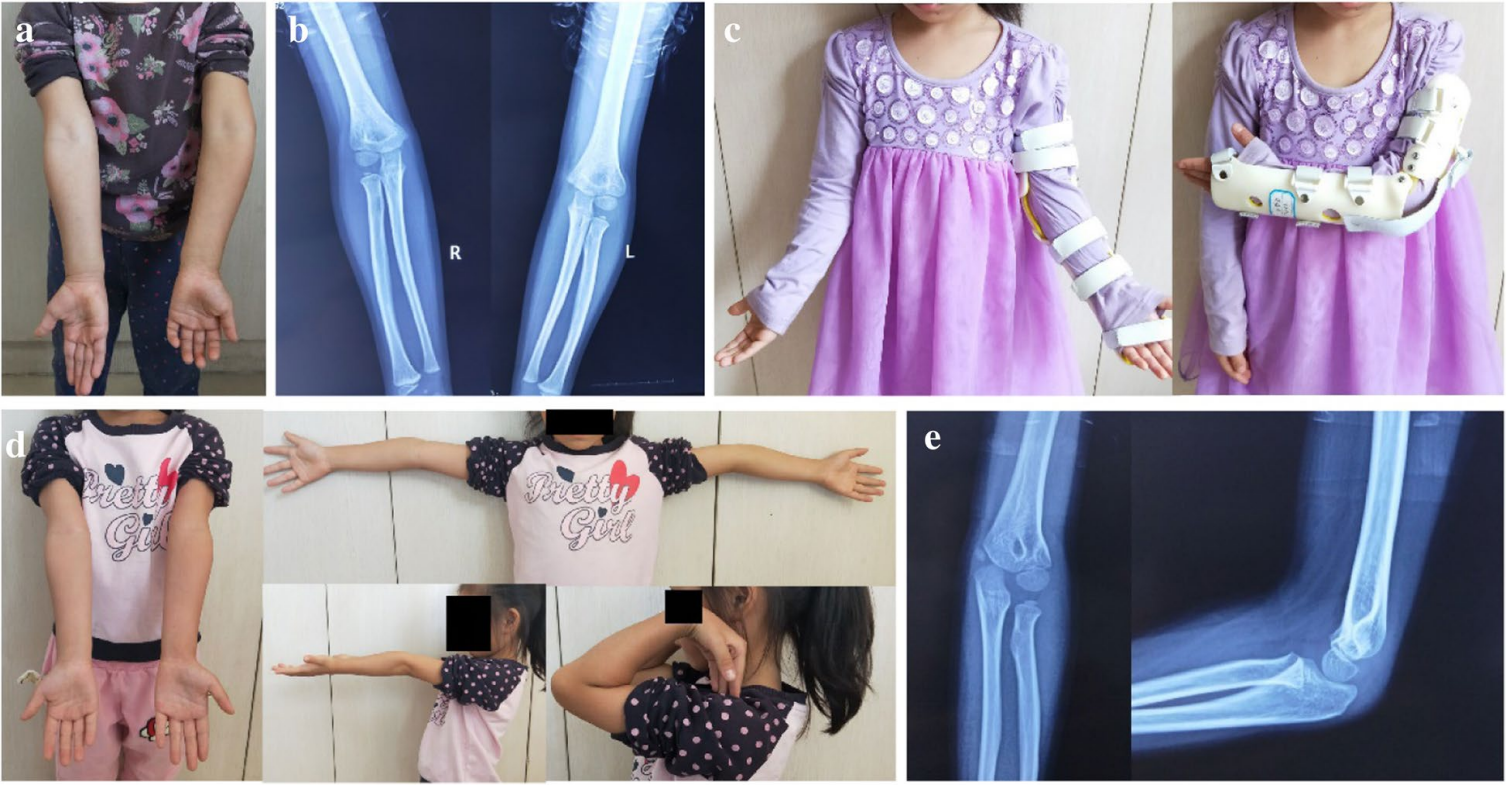
Case 1: **a** Pre-operative clinical appearance of bilateral upper limb (left side cubitus varus deformity). **b** Pre-treatment imaging. **c** Patients with cubitus varus wearing the functional brace. **d** Clinical appearances of bilateral upper limb. **e** Post-treatment radiographs of bilateral upper limb at last follow up

**Fig. 4:**
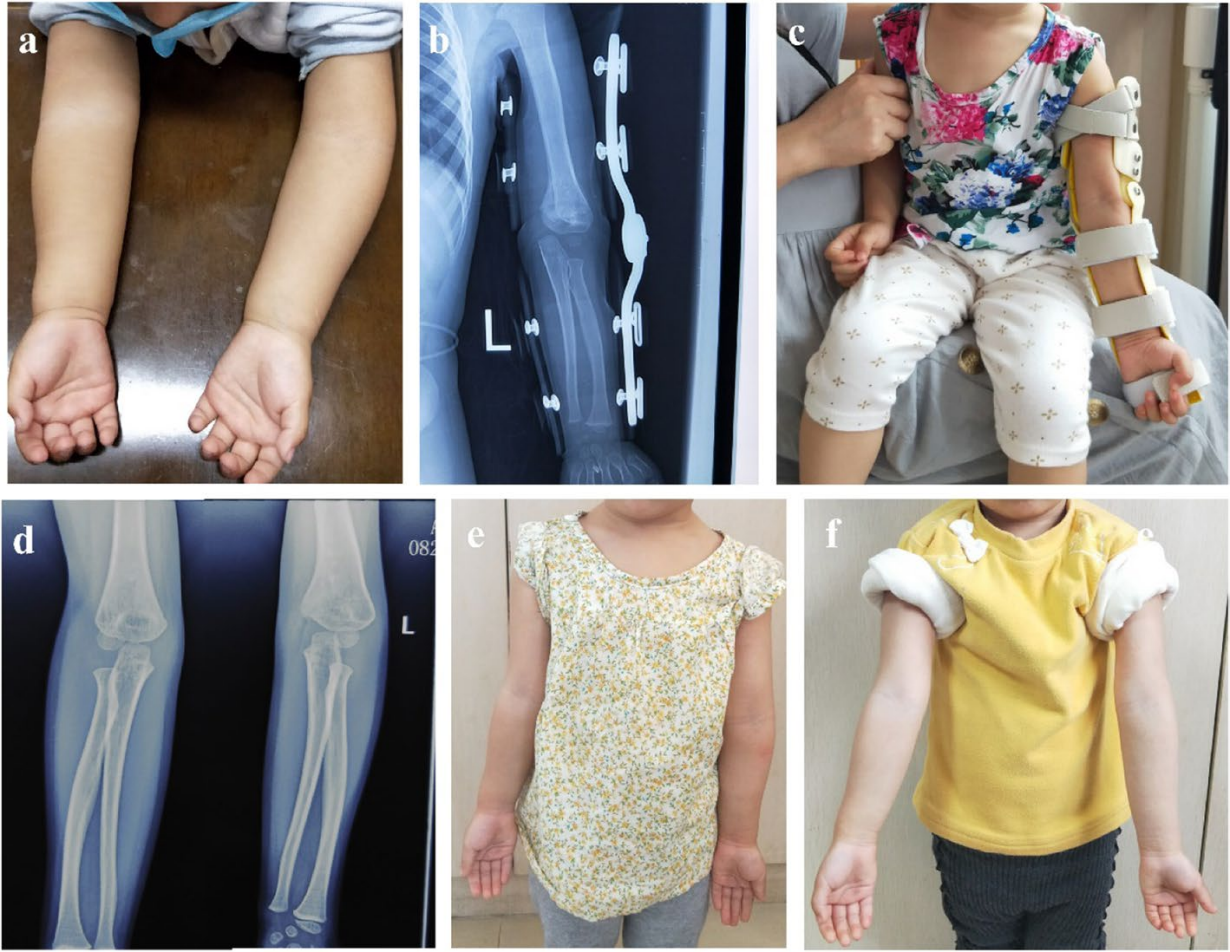
Case 2: **a** Pre-treatment clinical appearance of left cubitus varus deformity. **b,c** X-ray and clinical appearance after wearing the functional brace, respectively. **d** Post-treatment radiograph of bilateral upper limb at 1 year. **e,f** Clinical photographs of bilateral upper limb at 1 year and last follow up, respectively

No major complication or recurrence of deformity about FBPT was found in any case at the last follow-up. According to the Bellemore criteria, we got excellent results in fourteen patients (77.8%), good results in three patients (16.7%), and poor result in one patient (5.5%). Excellent and good results were considered satisfactory. There was no statistically significant difference between pre-treatment and post-treatment range of motion (*P* > 0.05). All patients and their parents (except one patient with residual varus deformity) were satisfied with the functional and cosmetic outcomes. The pre- and post-operative radiological and clinical images are shown in Figs. 3 and 4.

## Discussion

The results of the present study demonstrate that the majority of cubitus varus deformities can be treated nonsurgically for young children within one year after the initial injury using the FBPT technique.

Cubitus varus deformity is generally due to medial displacement or insufficient reduction of the distal humeral fragment after supracondylar humerus fractures, which remains a challenge for orthopedic surgeons [16]. Although many authors recommended early correction of pediatric cubitus varus deformity, the optimal timing has not yet been well-established in the literature [17–21]. Meanwhile, the ideal technique for cubitus varus correction remained controversial. Various osteotomies have been described and complications associated with these procedures include pin-tract infection, overcorrection or under-correction, prominence of the lateral condyle, and iatrogenic neurological injury [9, 22]. To the best of the authors’ knowledge, the spontaneous correction of the cubitus varus is highly unlikely to happen with time in growing children. Development in surgical techniques and orthopedic implants have resulted in a significant increase in the surgical treatment of cubitus varus. However, most of the patients and their parents are willing to obtain nonsurgical correction of cubitus varus for cosmetic reasons [23].

The Hueter-Volkmann law, which states that excessive pressure to a part of the joint leads to local growth retardation and reduced pressure to a part of the joint leads to local growth acceleration, explains why the FBPT technique can be successfully applied for children with cubitus varus [24]. When the band from the back was tightened, the brace can produce valgus stress on the extended elbow joint. After wearing 23 h a day for the first three months, it could provide constant compression on the lateral epiphysis of the affected elbow joint and then lead to growth retardation. For the medial epiphysis of the affected elbow joint, decreased stress leads to increased growth. Meanwhile, physical therapy was needed in all the patients in our series, which can enhance the effect of correction and avoid elbow joint stiffness or amyotrophy. Due to the slow and long-term treatment process, the patients need to return to the hospital regularly for observation and adjust the wearing time of the brace with high adherence.

In the present study, fourteen patients (77.8%) achieved excellent results while three patients (16.7%) achieved good results. On the other hand, the results of the meta-analysis for surgical treatment estimated an 87.8% overall rate of good to excellent results throughout the literature [20]. Therefore, the FBPT technique for cubitus varus conservative treatment achieves a higher rate of satisfactory results. The HEW angle was significantly improved from mean -23.2° (range, -38° to -12°) pre-treatment to mean 8.8° (range, -10° to + 15°) post-treatment. The advantages of FBPT technique include better correction and cosmetic outcome, no further surgery for implant removal, no complications such as scarring or lateral condyle prominence.

The limitations of this retrospective study include the small number of cases with a short follow-up period. Meanwhile, no comparison was made with other established techniques. This study is a single group, pre-post test design with no control group. Therefore, we will plan to conduct multi-center prospective study, involving a randomised controlled trial design to increase the internal validity of these results in the next step. Second, post treatment data including one month, three months, six months, 12 months and every year thereafter assessments are insufficient. Besides, cubitus varus is a 3D-deformity and therefore sagittal plane results via the FBPT should be included. Despite these limitations, this technique could provide a simple and reproducible therapeutic procedure for the correction of cubitus varus deformity in the clinic.

## Conclusions

In this study, our results suggest that FBPT is potentially effective for the treatment of cubitus varus for young children within one year after the initial injury. It could be a safe alternative to achieve better functional and cosmetic results without neurological injury or prominence of the lateral condyle, which could also prevent long-term sequelae such as ulnar neuritis, posterolateral rotatory instability, or chronic elbow pain.

## Data Availability

All data supporting our findings are contained within the manuscript.

## Abbreviations

3D: Three-dimensional
FBPT: Functional brace in combination with physical therapy
HEW: Humerus-elbow-wrist
